# Puberal and Adolescent Horse Riders’ Fitness during the COVID-19 Pandemic: The Effects of Training Restrictions on Health-Related and Functional Motor Abilities

**DOI:** 10.3390/ijerph19116394

**Published:** 2022-05-24

**Authors:** Sabrina Demarie, Emanuele Chirico, Cecilia Bratta, Cristina Cortis

**Affiliations:** 1 Department of Movement, Human and Health Sciences, University of Rome “Foro Italico”, Piazza de Bosis 6, 00135 Rome, Italy; e.chirico@studenti.uniroma4.it; 2 Department of Human Sciences, Society and Health, University of Cassino and Lazio Meridionale, 03043 Cassino, Italy; cecilia.bratta@studenti.unicas.it (C.B.); c.cortis@unicas.it (C.C.)

**Keywords:** SARS-CoV-2, adolescent fitness, young athletes, outdoor sport, horse riding

## Abstract

The aim of the study was to analyse the fitness level of young horse riders before and after 12 weeks of training restrictions instituted due to the COVID-19 emergency. Anthropometrical measure assessment and an eight-items fitness test battery were administered to 61 puberal and adolescent female amateur horse riders. Subjects were evaluated within 3 weeks before (pre-tests) the period of training restrictions and on the first day of normal training after it (post-tests). Post-test results showed significant increases in body weight (Z: −1.732; *p* value: 0.001; ES: −0.157) and BMI (F: 9.918; *p* value: 0.003; ES: 0.146), whilst the performance in hand grip and abdominal strength, hip mobility, and 10 × 5 m Shuttle and Cooper 12 min tests’ outcomes significantly decreased (F: 29.779; *p* value: 0.001 F: 29.779; *p* value: 0.001 F: 29.779; *p* value: 0.001 F: 29.779; *p* value: 0.001 F: 29.779; *p* value: 0.001, respectively). Correlation analysis revealed that riders’ experience was significantly correlated with hand grip (*p* < 0.01), leg strength (*p* < 0.01), hip mobility (*p* < 0.05), and 5 × 10 m Shuttle (*p* < 0.01) and the Cooper 12 min (*p* < 0.01) test results. It could be suggested that equestrian activities could produce a higher fitness level in puberal and adolescent riders, whilst home-based, unsupervised, and unattentively planned training during the twelve weeks of training restrictions might be insufficient to maintain it.

## 1. Introduction

Evidence that programmes promoting organised physical activities and sports amongst children and adolescents may contribute to the improvement of health-related quality of life has been systematically reported [1,2,3,4,5,6,7,8]. Horseback riding can be seen as an appropriate organised recreational physical activity, and indeed, it is practised by many young people. It is a sport which requires a high level of dexterity and postural muscular effort, with an emphasis on excellent fine motor skills and balance, in order for the rider to practise an ethological equitation [9]. It recruits aerobic and anaerobic energy systems with increasing energy cost as the horse progresses through the gaits; it improves postural control by coordinating one’s body movements with those of the horse, and it has been proven to produce effective physical fitness changes ranging over various aspects such as muscle strength and muscle mass, total and regional body composition, cardiorespiratory endurances, agility, and balance enhancement [10,11,12,13,14,15,16]. Amongst the most suitable equestrian disciplines for children and adolescents are endurance, pony games and show jumping. Endurance is a long-distance competition against the clock which tests the speed and endurance of a horse and challenges the rider’s effective use of pace. It requires a synergetic horse and rider combination to successfully complete a marked course within a specified time that is specifically designed to test their stamina and fitness on the track with distance, terrain, climate, and clock constraints without compromising the welfare of the horse. Although the rides are timed, the emphasis is on finishing the race in good condition rather than coming in first. Each course is divided into phases with a compulsory halt for a veterinary inspection which determines whether the horse is fit to continue or not. At the beginner’s level, horses complete courses that vary from 15 km to 19 km long, either at walking or trotting speed. Pony games is a team sport that combines the love of ponies and friends with various races. Teams of four or five riders and ponies take part in a series of exciting races that involve a mix of turns, handovers, skill, vaulting, and galloping against other teams. Competitions are comprised of several relay-style races, requiring riders to pick up objects from the ground while remaining in the saddle, weave through a series of poles at high speeds, hand items to teammates without slowing their ponies, and dunking objects into buckets; beginners usually compete at the walking and trotting gaits. Show jumping is a competitive equestrian sport in which the horse and rider are required to jump over all of the fences in a course that has been designed for a particular show without knocking them down and within a set time limit. Competitors compete one by one, and judges determine whether the fences are successfully cleared and assign penalties and points; then, the times and scores are compared to determine the winner.

In Italy, where the study was conducted, after the first period of the SARS-CoV-2 pandemic when physical activities were restricted to home-based training and individual outdoor jogging and running, competitive horse riders have attempted other forms of training besides those on the horse; nonetheless, they suffered from significantly decreased performance with a substantial contribution of cognitive distress to the overall perception of effort [17]. It is worth noting that in Italy, high-level athletes and horse owners have been allowed to resume training after eight weeks of lockdown, while recreational athletes were kept from sport activities for four weeks longer. This led to concerns about the impact of detraining on young athletes’ health, performance, and injury risk at the resumption of regular physical activity [18,19,20].

To this purpose, a detailed profile of athletes’ physical fitness allowed not only the determination of the underlying performance qualities for training-planning purposes, but also a wide range of health-related attributes associated with the quality of life. Many studies investigated fitness- and health-related changes after the COVID-19 confinement in healthy or diseased school children and adolescents [21,22]. However, young athletes could suffer a major impact during confinement due to their greatly modified lifestyle. Most athletes will find themselves searching for an optimal solution to maintain their physical, physiological, and psychological levels as close as possible to their original ones. Many studies collected questionnaires and interview data on young athletes’ confinement and on its outcomes, and general guidelines have been provided for athletes to optimally maintain physical and mental fitness whilst respecting the measures taken during the COVID-19 confinement period [23]. However, to the best of our knowledge, no studies collected objective measures of physical activity and fitness level changes in young, healthy athletes during the first strict confinement period that could be useful for planning the activities to be implemented in youth sports settings if a new wave of confinements were to occur in the future.

To quantitatively assess the physical attributes of the young and children, field fitness test batteries can be administered with inexpensive and easy-to-use equipment, being therefore useful for the continuous monitoring of training effects and fitness levels as well as for the comparison amongst different populations [24,25,26].

To assess the effect of twelve weeks of training restrictions due to the COVID-19 pandemic on young recreational horse riders of different equitation disciplines, the aim of the study was to analyse their fitness level by administering a motor fitness test battery before and after the emergency period and to compare their results with reference values of healthy, active, age-matched athletes of the same geographical region.

## 2. Materials and Methods

Sixty-six female puberal and adolescent (age range 9 to 18 with a mean age of 13.87 ± 0.34) recreational horse riders practicing endurance, pony games and show jumping equestrian disciplines were recruited for the study. Inclusion criteria were participating regularly and continuously and taking equestrian classes at least twice a week for 12 or more months. Exclusion criteria were noncomplying with home-based training during all of the 12 weeks of training restrictions, testing positive for SARS-CoV-2, or suffering injuries or illnesses during the test period or in the 3 preceding months. Three of the subjects either tested positive for SARS-CoV-2 or suffered injuries or illnesses during the test period or in the 3 preceding months, and 2 of them did not practice horse riding continuously in the previous year. This led to the exclusion of 5 riders and left 61 participants equally distributed amongst the three equestrian disciplines (Endurance, *n* = 20; Pony Games, *n* = 20; Show Jumping *n* = 21). Participants’ equitation experience was assessed as the number of months ranging from the first time they rode a horse to the pre-test date, with a mean value of 13 ± 3.1 months. During the 6 months before the study, the subjects had regularly completed 2 ± 0.3 training sessions per week, each lasting 45–50 min each on the horse.

All riders and their legal tutors received written and oral information about the purpose of the study, of their rights as study participants, and of the anonymity of their data and provided written informed consent. The project received approval from the institutional review board (protocol code CAR-14/2019).

**Figure 1:**
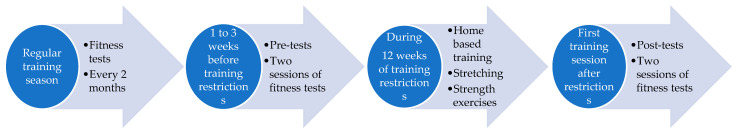
Training and test sessions timeline.

As depicted in Figure 1, fitness test batteries were part of the regular training program and were usually administered every 8 weeks by the coaches of the clubs involved. Since subjects were familiar with all test protocols long before data collection, a learning effect impact on the post-test outcome should not be likely. Tests were administered throughout the 3 weeks preceding the pandemic period and immediately after the resumption of physical activity after the 12 weeks of training restrictions. The motor fitness test battery comprised 8 items performed as previously described [24,26].

**Figure 2:**
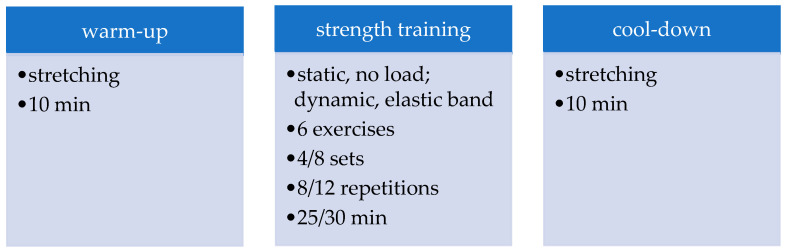
Description of the home based training sessions.

During the 12 weeks of the training restriction period, athletes trained on their own, on a regular basis, for approximately 45–50 min 2 days per week; the routine comprised stretching warm-up, no-load isometric and elastic-band-resisted exercises for the core, and upper and lower limb muscle strength training, as depicted in Figure 2. Home-based training was initially programmed for 8 weeks; one set and one repetition were added for every exercise every 2 weeks. From the first to the eighth and from the nineth to the twelfth week, the training programme remained unchanged [27].

Compliance with the home-based training programme was ascertained by text messages to the coaches listing the number of exercises, sets, and repetitions completed during each week. The weekly training programme was considered completed if at least 80% of the maximal repetitions and sets were accomplished for each exercise.

### 2.1. Anthropometric Measures

To measure height, the athlete stood on the stadiometer platform with their shoes off. Their feet were together, and the heels were supported on the base of the device with the arms along the body. The participant was instructed to gaze outward, and the measurement was rounded to the nearest 0.5 cm. We assessed the body weight simultaneously by instructing the athlete to step on the scale (with their shoes off). The results were recorded in kilograms and centimetres (HE HEM Medical Weight Scale with Stadiometer, HIWEIGH, Shanghai, China). All anthropometric measurements were taken by the same medical doctor in charge of all of the clubs involved. Body mass index (BMI) was calculated as weight in kilograms/height meters squared [28].

### 2.2. Motor Fitness Test Batteries

Motor fitness tests are comprised in the Eurofit and ALPHA test batteries that have been widely used throughout Europe with children and adolescents [29]. They have been developed as a standardised European fitness test battery used to assess the effectiveness of physical education and to measure the health-related fitness of schoolchildren [30]. The fitness test battery’s inter-rater reliability and test-retest reliability are considered adequate to support their clinical use; indeed, they have been successfully used with primary, middle, and high school students, providing reference data bases [26,31]. The same test items have been used in the school districts of our children and adolescents, ensuring that subjects may have come from similar ethnic, cultural, socio-economic, and socio-demographic groups. Physical education schoolteachers provided the results of their last collection, which served as reference values for comparison with our subjects.

The sit-and-reach test was employed to assess hip mobility since it has been suggested that hip flexibility is the main determinant of the back-saver sit-and-reach test in adolescents, and no significant differences in inter-method agreement were observed between the back-saver sit-and-reach test and the sit-and-reach test, implying that they are comparable [32]. The flamingo test was demonstrated to be suitable for measuring youth aged 9 to 17 years [33]. Amongst the balance tests, the flamingo test results were mostly predicted by strength of the abdominal muscle in pre-adolescent girls [34]; therefore, it seemed to be the most suitable for horse riders that centre their motor control in the core area. Moreover, the flamingo test showed the highest associations with the other motor abilities and correlated the best with endurance running as compared to the one-leg stance on a low beam and the low-beam walking test. Amongst the three tests, it also showed the highest reliability score (0.910) in pre-adolescent children [35].

In the flamingo test for balance, participants stood upright on a special wooden beam (50 × 3 × 4 cm). The leg they stood on was fully extended, whilst the free leg was bent at the knee, and the foot of that leg was held with the hand on the same side of the body. The timekeeper helped the participant assume the correct position and started taking the time when the subject released the timekeeper’s hand. The result was the maximum number of attempts in 1 min, which was limited to 30. If the subject exceeded this number 15 times in the first 30 s, the subject’s result was recorded as 31 [35].

The sit-and-reach test required the use of the sit-and-reach standardized box; the participant was required to sit with their knees straight and legs together, and their feet placed against the box, then they slowly reached forwards as far as possible and the furthest position the participant could reach (in centimetres) was recorded [32].

Hand grip was tested for the dominant hand only using a digital hand dynamometer (TKK 5101 Grip-D, Takey, Tokyo, Japan). The subjects looked forwards with their feet shoulder-width apart and were instructed not to touch the dynamometer with any part of the body except for the hand being measured. During the test, subjects stood with their arm straight down at their side, their shoulder slightly abducted (approximately 10°), their elbow in full extension, their forearm in a neutral position, and their wrist extended; the display of the dynamometer was aligned so it would face the examiner, providing blind measurements to the subject. Participants were instructed to squeeze gradually and continuously for at least 2 s and were encouraged to do their best when performing the tests [36].

For the standing broad jump for leg strength, the participant stood behind the starting line with their feet together and pushed vigorously in order to jump forwards as far as possible. The distance was measured with a 10 m line (echoENG, Cormano, Italy) from the take-off line to the point where the back of the heel was closest to the take-off line after landing on the mat or anti-slip floor [37,38].

Sit-ups were used to test abdominal strength and required the subjects to lie face-up on a mat in a supine position with their knees bent at 90-degree angles. The feet were placed flat on the mat and held in position by the examiner. The subjects’ arms were crossed on their chest with the hands on the opposite shoulders. When the examiner signalled the start, a timer was started, and the subjects performed as many repetitions as they could within 30 s. To complete a full repetition, each subject flexed their trunk, allowing their lower back to come off the mat, until the subject’s elbows contacted his or her thighs. This movement was reversed to the starting position, and the sequence was repeated until the 30 s had expired. The examiner counted the number of repetitions, and the passing of the 30 s was assessed with a stopwatch (Hanhart Delta E 200 1/100 sec., Hanhart, Gütenbach, Germany) [39,40].

For the flexed-arms hang tests that tested the arms’ strength, participants were asked to hang for as long as possible on a horizontal bar with a flexed arm so that the chin was level with the horizontal bar [41]. The subject was assisted into position, and the bar was grasped using an overhand grip with the hands shoulder width apart; the timer started when the subject was released. The timer was stopped when the subject’s chin fell below the level of the bar or the head tilted backwards to enable the chin to stay level with the bar. The time was recorded using a stopwatch (Hanhart Delta E 200 1/100 sec., Hanhart, Gütenbach, Germany).

In the 10 × 5 m shuttle test for speed and agility, marker cones were placed five metres apart, and participants were required to run back and forth between them for a total of 50 m. They started with a foot on one marker; when instructed by the timekeeper, the subject ran to the opposite marker, turned, and returned to the starting line. This was repeated five times without stopping. At each marker, both feet were required to fully cross the line, and the time it took to cover the 50 m was measured with a stopwatch (Hanhart Delta E 200 1/100 sec., Hanhart, Gütenbach, Germany) [42].

In the Cooper’s 12-min run test for aerobic fitness, the distance travelled in this period was measured. The test was performed on an athletic track by counting the number of laps in the established perimeter with a known distance. The measurements of the perimeter and the distance of the incomplete turns were measured with a 50 m line (echoENG, Cormano, Italy) and the passing of the 12 min was assessed using a stopwatch (Hanhart Delta E 200 1/100 sec., Hanhart, Gütenbach, Germany) [43].

Tests were divided into two separate sessions. Session 1 comprised the Cooper’s 12-min run, the sit-and-reach, the hand grip, and the standing broad jump tests. Session 2 comprised the 10 × 5 m shuttle, the flamingo, the sit-ups and the flexed-arms hang tests. The running test was completed at the end of the test session; the others were performed in a random order by each subject.

All tests were preceded by a 15 min warm-up consisting of running and stretching for both the upper and lower limbs as well as for core activation.

For each item, the best of 3 attempts was retained, except for the 10 × 5 m shuttle and the Cooper 12 min tests, which were performed once for the pre-test and once for the post-test. All the tests were administered by expert coach graduates in sports science; the same coach oversaw the tests both in the pre- and the post-pandemic periods.

### 2.3. Reference Fitness Test Values

Fitness test batteries have been applied in the school districts of our group of puberal and adolescent horse riders, ensuring that subjects may have come from similar ethnic, cultural, socio-economic, and socio-demographic groups. Physical education schoolteachers provided us with the results of their last collection of fitness values dated 2007. The results for the same 8 test items obtained from 1687 female high school non-athlete students were used. Since these values are age- and sex- matched (age: 11–18 years), they served as reference values to be compared with those of our subjects.

### 2.4. Statistical Analysis

For all data, descriptive statistics were calculated, and distributions were verified to identify potential outliers. We also checked the distribution to see if assumptions of normality had been violated by a Shapiro–Wilk test. In the case of skewed distribution, differences between the pre- and post-tests were analysed through a Mann–Whitney U as a non-parametric method. For variables that were normally distributed, we applied a repeated measure ANOVA and Bonferroni post hoc. This statistic model was chosen to evaluate the differences within the groups (pre and post phases) and the differences between the groups, specifically the three different equitation disciplines. Differences between endurance, pony games, and show jumping were tested through a Kruskal–Wallis test as a non-parametric method and by the Bonferroni post hoc test of repeated measure ANOVA in the case of normally distributed variables. To assess the differences between horse riders and age-matched reference values, an independent simple *t*-test was utilised. To understand possible relationships between riding experience and pre-test measured variables, Spearman rank correlation or Pearson correlation analyses were applied when proper. Significance was accepted at the level of *p* < 0.05.

## 3. Results

Height, BMI, flamingo test, abdominal strength, 5 × 10 m shuttle, and hip mobility presented a normal distribution of data, while age, riding experience, weight, hand grip, leg strength, arm strength, and the Copper 12 min test did not.

The comparison between pre and post anthropometrical measures revealed that the mean body weight and BMI significantly increased during the twelve weeks of training restrictions, by 3.9% and 8.5%, respectively, as shown in Table 1.

As reported in Table 2, hand grip, abdominal strength, and hip mobility results significantly reduced after the training restrictions period—by −15.4%, −6.9%, and 82.9%, respectively. Furthermore, the 5 × 10 m shuttle, and Cooper 12 min test results also appeared significantly diminished in the post-test—by −3.5% and −11.5%, respectively.

In Table 3, riders’ results collected in the pre-tests are confronted with age- and sex-matched reference values obtained by a report on 1687 female middle and high school students (age: 11–18 years) of the same school district. Riders’ body weight results were significantly lower (−5.6%) than age-matched non-athletes’. Pre-test fitness measurements were significantly lower except for handgrip, which was significantly higher (17%).

Riders of each discipline did not present significant differences in anthropometrical characteristics. The subjects were 14.3 ± 2.9, 13.2 ± 2.1, and 14.1 ± 3.0 years old; their heights (cm) were 161.1 ± 8.5, 155.3 ± 9.3, and 162.0 ± 8.1; their weights were (kg) 50.6 ± 9.3, 46.0 ± 7.0, and 50.8 ± 6.7; and their BMIs were 19.4 ± 2.5, 18.5 ± 2.2, and 19.3 ± 1.6 for endurance, pony games and show jumping, respectively.

Differences amongst equestrian disciplines highlighted that the endurance group (Table 4) presented the statistically lowest abdominal strength and the show jumping group showed the strongest hand grip values and the statistically highest arm strength in comparison to the other two disciplines.

Correlation analysis revealed that riders’ experience was significantly correlated with hand grip, leg strength, hip mobility, and with the 5 × 10 m shuttle and the Cooper 12 min test results, as reported in Table 5.

## 4. Discussion

Results of the present study evidenced that twelve weeks of training restrictions due to the COVID-19 emergency and lockdown decreased the fitness level of young recreational horse riders. Even though pupils were instructed by coaches to practise home-based physical activities, it can be argued that either general and unguided exercise assignments of 45–50 min 2 days per week were insufficient to retain adolescent physical fitness or that assignments’ compliance was not respected, if not both. It has been demonstrated that more experienced athletes of the same regional area (4 years of experience or more) who have had the opportunity to be remotely assisted, guided, and monitored by their coaches were more engaged in home training than their recreational counterparts, being able to keep high physical activity levels even in an extraordinary situation such as a nationwide lockdown [44]. It can be suggested that during training restriction periods, recreational athletes may need more thorough fitness programs with more detailed instructions and stricter surveillance than high-level athletes in order for home-based training to be effective. Moreover, due to the possibly more pronounced detraining effect on novice and recreational than on professional athletes, their training resumption should be even more carefully managed before returning to full training intensities and volumes.

Anthropometric measurements of our group of young riders showed no differences in height but significant increases in body weight and BMI. It can thus be argued that equitation training apparently allowed the subjects to keep weight and BMI under control before training restrictions intervened. Since BMI is generally related to an increased risk of various diseases such as type 2 diabetes and heart disease and can have an important influence on adults’ health conditions, horse riding exercises can be suggested to be associated with people’s health management [45].

It has also been put forward that, together with the athlete’s physical fitness, their mental well-being can be affected by training restrictions [46,47,48]. The role of psychological components in equitation disciplines is considered vitally important and athletes are required to always be in control of both their body and mind [49,50,51]. The cooperative effort of two non-related species, horses and humans, is essential to continually adapt to various unpredictable situations. The horse’s nature is governed by its instinctive reactions, by the different gait employed, and by the characteristics of the land where the practice is being carried out. Hence, a positive interaction between the horse and its rider when coping with the emotional and physical challenges of equestrian tasks is a prerequisite for success not only for competitions, but also for recreational activities [10,52]. Further supporting this, in competitive horse riders, 8 weeks of training restrictions decreased the performance outcome for up to six weeks following training resumption with a significant rise in anxiety and rate of perceived effort during competitions [17]. Athletes with higher levels of anxiety, such as those who do not respect sufficient recovery times, can suffer a higher risk of injuries [53,54]. When training restarts, great attention should be paid not only to the training workload but also to athletes’ physical and mental conditions, perception of exertion, signs, and symptoms of anxiety [55]. For a safer and more efficient way to resume training, young novice riders can begin practising in conditions of reduced anxiety, such as on mechanical horse simulators [56,57]. Such simulators have been proven to be effective and sufficient indoor workout equipment for people with limited time and chances for outdoor activities and for enhancing neurologic functions in patients [6,58,59]. A study aimed to examine the energy expenditure and postural coordination of horse riders and non-riders on a mechanical horse indicates a change in the energy system from an aerobic mode at a low oscillation frequency to a lactic anaerobic mode at a high oscillation frequency for both groups [57]. Horse riding simulation training can thus be a fun and interesting alternative practice tool to resume equitation practice which allows the avoidance of the interference of emotional distress, which may increase the fitness level and the motivation to participate in exercise programmes.

Concerning horse riding training effects on physical fitness, medium-to-high training loads in various equitation disciplines have been reported for general competitive riders, for college females, for sedentary young female adults, and for healthy children, suggesting that it is possible to achieve health benefits through accumulated horseback riding exercise, particularly if riding is performed at the more intense gaits [6,52,60,61,62,63,64,65]. Olympic equestrian athletes have been reported to have high values of muscle strength and balance, good physical functions, and good maximal aerobic power [16]. Results of the present study on recreationally trained young horse riders indicated higher hip mobility (−82.8%) and hand grip (17%) with respect to age-matched reference values of the same geographical region. The latter could be expected since the greater effort in horse conduction relies on the upper limbs and hands, particularly in novice riders that depend on rough and taut controls of the horse, with the use of excessive muscular force of the arms and hands [63]. This is also confirmed by the significantly higher arm strength showed by the show jumping group, being the discipline that requires the most directional control by the riders as compared to endurance and pony games. High hip mobility can be explained by the fact that all coaches participating in the study included mobility exercises in their usual sessions, which is not a common practice of horse riding trainers.

On the other hand, our fitness test results indicated lower values, except for handgrip, than those obtained by teachers in the same school district from age- and sex-matched non-athlete students that we used as reference. However, it must be noted that reference values were collected fifteen years earlier and could be irrelevant for the present puberal and adolescent population [30]. Indeed, accelerometery data show that sedentary behaviours of adolescent girls (12–15 years old) are increasing over time from 2003, with higher rates reported in recent studies [66]. Consistently, in recent years, two thirds of European children and adolescents were categorized as not sufficiently physically active, with lower physical activity levels in Southern European countries and girls who are less active and more sedentary in all age categories [67]. Therefore, outdated values referring to a possibly more active population of the past may lead to an underestimation of the horse riding training effect on the contemporary young population. Indeed, values measured after the 12 weeks of forced restriction from equitation training indicated a significant loss of hand grip and abdominal strength and 5 × 10 m shuttle, and Copper 12 min test results and a significant increase in body weight and BMI. Moreover, our results showed that more experience in horse riding was significantly correlated with higher hand grip, leg strength, and hip mobility, and with the better results in the 5 × 10 m shuttle test and the Cooper 12 min test. Therefore, recreational equitation practice could be presumed to be a physical activity that offers some training effects suitable for improving the fitness level of young recreational riders.

With regards to the equestrian discipline that best suits fitness enhancement purposes, values of the three groups are consistent between them, indicating similar training effects. However, endurance riders appeared to achieve higher aerobic fitness involvement and lower abdominal strength, whilst show jumping riders showed the highest hand and arm strength. This could be ascribed to their opposite metabolic requests and different needs for precise guidance of the horse. It can thus be supposed that each of the two disciplines can be more suitable than the other if either cardiometabolic fitness or muscle strength is the main goal, whilst pony games could be envisioned as the least specialised equitation activity out of the three.

Limitations of the present study are represented by the lack of a control group, which does not allow us to determine if pre-test fitness levels were attributable solely to horse riding or if other aspects might have been involved. Additionally, during training restrictions, physical activity was entrusted to individual responsibility, giving rise to different sorts of fitness outcomes. Further limitations were the small number of subjects for each equitation discipline, the all-female subjects, and the sample of items chosen to be part of the test battery that could not represent the actual horse riders’ training effects. It could certainly be useful to discriminate which physical attributes are most pertinent to equitation training and performance by broadening the motor fit test items and by correlating test results with performance outcomes.

## 5. Conclusions

Twelve weeks of training restrictions due to the COVID-19 sanitary emergency decreased the fitness level of puberal and adolescent female recreational horse riders. It could thus be suggested that home-based, unsupervised, and unattentively planned training can be insufficient to maintain their fitness level during home confinement.

## Figures and Tables

**Table 1:** Anthropometric measures collected before (pre-test) and after (post-test) the twelve weeks of training restrictions.

	Age (years)	Height (cm)	Weight (kg)	BMI
Pre-test	13.9 ± 2.7	159.4 ± 9.0	49.1 ± 7.9	19.1 ± 2.1
Post-test	13.9 ± 2.6	159.4 ± 8.9 *	51.0 ± 7.5	20.7 ± 3.4 *
F		29.779		9.918
Z	−1.732		−1.732	
*p* value	0.083	0.083	0.001	0.003
Effect size	−0.157	0.997	−0.157	0.146

* Significant differences between pre- and post-test results, *p* < 0.05.

**Table 2:** Motor fitness test battery results collected before (pre-test) and after (post-test) the twelve weeks of training restrictions.

	Hand Grip (kg)	Flamingo Test (s)	Legs Strength (cm)	Abdominal Strength (n)	Arms Strength (s)	5 × 10 m Shuttle (s)	Cooper 12 min (m)	Hip Mobility (cm)
Pre-test	29.3 ± 5.7	9.5 ± 4.2	146.0 ± 17.4	18.3 ± 3.8	10.2 ± 3.9	23.0 ± 1.9	1282.9 ± 196.8	1.1 ± 7.3
Post-test	24.8 ± 3.0 *	9.2 ± 4.7	144.2 ± 21.0	17.0 ± 3.1 *	9.5 ± 2.7	23.9 ± 2.2 *	1135.4 ± 149.3 *	2.1 ± 7.2 *
F		0.135		13.074		15.725		11.732
Z	−4.353		−0.779		−1.219		−4.816	
*p* value	0.000	0.714	0.436	0.001	0.223	0.000	0.000	0.001
Effect size	−0.384	0.002	−0.071	0.184	−0.110	0.213	−0.436	0.168

* Significant differences between pre and post-tests results, *p* < 0.05.

**Table 3:** Comparison of the fitness pre-test horse riders’ results with reference values.

	Age (yrs)	Height (cm)	Weight (kg)	BMI	Hand Grip (kg)	Flamingo Test (s)	Legs Strength (cm)	Abdominal Strength (n)	Arms Strength (s)	5 × 10 m Shuttle (s)	Cooper 12 min (m)	Hip Mobility (cm)
Reference values	13.9 ± 2.7	158.2 ± 7.6	52.0 ± 7.4	19.1 ± 1.6	25.1 ± 4.0	10.7 ± 1.2	154.9 ± 7.6	19.3 ± 0.9	17.8 ± 2.8	21.0 ± 0.1	1743.1 ± 43.8	6.7 ± 1.9
% diff.	0.4	1.20	−5.6	−0.3	17.0	−11.2	−5.8	−5.3	−42.8	9.7	−26.4	−82.8
Z	0.000	−0.311	−2.636	−0.110	−4.236	−2.079	−3.857	−1.974	−8.399	−6.799	−9.547	−5.499
*p* values	1.000	0.756	0.008 *	0.912	0.000 *	0.038 *	0.000 *	0.048 *	0.000 *	0.000 *	0.000 *	0.000 *
Effect size	0.001	−0.028	−0.239	−0.010	−0.383	−0.188	−0.349	−0.179	−0.760	−0.616	−0.864	−0.498

* Significant differences between horse riders’ results and age-matched reference values *p* < 0.05.

**Table 4:** Fitness values of riders for each equestrian discipline.

	Handgrip (kg)	Flamingo Test (s)	Legs Strength (cm)	Abdominal Strength (n)	Arms Strength (s)	5 × 10 m Shuttle (s)	Cooper 12 min (m)	Hip Mobility (cm)
Endurance	28.2 ± 5.9 *	10.9 ± 32.	150.1 ± 9.0	16.1 ± 3.4 *	9.3 ± 3.7 *	23.9 ± 1.4	1301.7 ± 216.1	1.3 ± 8.7
Pony Games	28.3 ± 5.4 *	9.3 ± 5.5	144.0 ± 24.2	19.3 ± 3.6 *	8.8 ± 0.3 *	23.4 ± 2.6	1316.0 ± 170.5	2.3 ± 7.1
Show Jumping	31.6 ± 5.4 *	8.4 ± 3.0	144.0 ± 15.2	19.5 ± 3.5 *	12.6 ± 4.4 *	24.4 ± 2.4	1229.3 ± 201.1	−0.2 ± 6.1
*p* value	0.023			0.008	0.002			

* Significant differences amongst the riders’ results of three equestrian disciplines, *p* < 0.05.

**Table 5:** Correlation coefficients of fitness values with months of equitation experience.

	Exp. (months)	Weight (kg)	Height (cm)	BMI	Handg. (kg)	Flamingo (s)	Abd. (n)	5 × 10 m (s)	Hip (cm)	Legs (cm)	Arms (s)	Cooper (m)
Age (y)	0.978 **											
Exp. (months)	1.000											
Weight (kg)	0.441 **	1.000										
Height (cm)	0.645 **	0.774 **	1.000									
BMI	−0.001	0.743 **	0.266 *	1.000								
Handg. (kg)	0.398 **	0.423 **	0.499 **	0.159	1.000							
Flamingo (s)	−0.093	−0.083	−0.098	−0.004	−0.364 **	1.000						
Abd. Str. (n)	0.075	−0.123	−0.109	−0.190	−0.068	−0.084	1.000					
5 × 10 m (s)	−0.343 **	0.013	−0.232	0.166	−0.226	0.209	−0.168	1.000				
Hip (cm)	−0.285 *	−0.141	−0.296 *	0.028	−0.259 *	0.211	0.013	0.194	1			
Legs (cm)	0.408 **	0.077	0.258 *	−0.085	0.126	0.025	0.219	−0.395 **	−0.083	1.000		
Arms (s)	0.139	0.108	0.234	−0.077	0.323 *	−0.187	0.229	−0.124	−0.304 *	0.256 *	1.000	
Cooper (m)	0.483 **	0.016	0.133	−0.181	−0.023	0.168	0.181	−0.429 **	−0.146	0.499 **	0.499 **	1.000

Age in years, Exp. (riding experience in months), Weight (in kilograms), Height (in centimetres), BMI, Handg. (hand grip in kg), Flamingo (balance test in seconds), Abd. Str. (abdominal strength test as number of repetitions), 5 × 10 m (10 × 5 m shuttle test in seconds), Hip (hip mobility tests in centimetres), Legs (legs strength in centimetres), Arms Str. (arm strength in seconds), Cooper (Cooper 12 min test in metres). * *p* < 0.05; ** *p* < 0.01.

## Data Availability

Not applicable.
